# Quadratic programming-based autonomous cruise control of an intelligent tugboat

**DOI:** 10.1371/journal.pone.0345699

**Published:** 2026-04-07

**Authors:** Hongkun He, Tingyu Peng, Haoze Li, Dazhi Huang, Cunlong Liu

**Affiliations:** 1 School of Ocean Engineering, Jiangsu Ocean University, Lianyungang, China; 2 Lianyungang Hongyun Co., Ltd, Lianyungang, China; Istinye University: Istinye Universitesi, TÜRKIYE

## Abstract

To address the multi-objective control problem of autonomous cruising, collision avoidance, and input constraints in intelligent tugboats, a quadratic programming-based autonomous cruise control method is proposed. The method enables the tugboat to reach the target location with prescribed speed, heading, and path while rigorously avoiding collisions within its actuation limits. First, a desired control input is derived using back-stepping and sliding mode control to ensure asymptotic stability of the tracking error. Second, based on Nagumo’s theorem, the positional constraints for safe collision avoidance of the tugboat are equivalently transformed into input constraints, effectively preventing any collisions with other vessels. Third, a unified controller is synthesized using a quadratic programming approach to optimally balance cruising control, collision avoidance, and input limitations. Finally, simulation results demonstrate that the proposed quadratic programming-based autonomous cruise control method enforces the prescribed safety distance and actuator limits, while avoiding large or persistent deviations from the reference trajectory and allowing the desired speed and heading to be re-established rapidly after the avoidance maneuver.

## 1. Introduction

As specialized marine support vessels, tugboats play a vital role in assisting large ships during berthing, unberthing, towing, and escort operations, particularly in confined waterways and busy port areas [1]. In such environments, tugboats often operate under highly congested traffic conditions, where even small control errors can lead to contact or collision incidents with significant safety and economic consequences [2]. Against this backdrop, rapid advances in intelligent technologies are accelerating the shift from manual to automated systems and motivating port authorities and operators worldwide to deploy intelligent and semi-autonomous tug solutions that enhance situational awareness and reduce human workload [3]. As a result, intelligent tugboats equipped with autonomous route planning, dynamic speed and heading adjustment, and constraint-aware obstacle avoidance are emerging, providing crucial support for high-frequency, repetitive tasks such as port operations and navigation in confined waterways.

Zhong et al. [4] developed a communication and navigation control system for unmanned surface vessels using fuzzy PID control, enabling basic autonomous cruising. However, their motion control strategy lacked adaptability to complex environments. Yu [5] proposed a ship trajectory tracking method based on deep reinforcement learning, combining line-of-sight guidance, Markov decision process modeling, and convolutional neural networks to optimize rudder angle control. Similarly, Zhang R et al. [6] designed a robust trajectory tracking scheme for USVs under dynamic constraints and environmental disturbances, utilizing virtual system modeling and a normalization-based backstepping controller to achieve high-precision performance. In fact, tugboats primarily operate in congested and narrow waterways with heavy traffic, whereas the above trajectory tracking strategies focus only on optimization in open-water scenarios and lack the ability to perceive and avoid dynamic obstacles on the water.

Zhao et al. [7] proposed an integrated collision avoidance and trajectory tracking solution using policy gradient reinforcement learning, achieving autonomous multi-vessel collision avoidance. However, the training and inference of the model require heavy computational resources, making real-time implementation challenging. Zhang Z et al. [8] developed a computer vision and LiDAR-based method for autonomous navigation and collision prediction in port waterways. By integrating artificial potential fields (APF) with an improved Rapidly-exploring Random Tree algorithm, they achieved significant improvements in navigation efficiency and collision prediction accuracy. Nevertheless, the APF-based strategy exhibits inherent conservatism and limited global perception, frequently resulting in local optima. These limitations hinder reliable long-term autonomous operations in complex environments. Tugboat operating environments are inherently complex and highly susceptible to collisions with other port vessels. Given the tightly scheduled and continuous nature of tugboat missions, the onboard collision avoidance algorithms must be real-time and precise.

Zhang K et al. [9] proposed an intelligent dynamic collision avoidance method that integrates trajectory prediction with an improved velocity obstacle algorithm, enabling real-time computation of avoidance maneuvers. Kalman filtering is employed to predict the motion states of surrounding vessels for dynamic obstacle avoidance. However, the method primarily targets avoidance behavior and lacks a systematic framework for multi-objective coordination and control. Huang et al. [10] developed a velocity obstacle-based anti-collision system that directly calculates ideal velocity adjustment paths, greatly enhancing computational efficiency and online decision-making speed. Yet, it ignores the physical limitations of vessel maneuverability, which may render the control strategy infeasible in practice. Menges et al. [11] proposed a nonlinear model predictive control method that combines APF with a nonlinear disturbance observer to simultaneously achieve collision avoidance, path tracking, and disturbance rejection. By enforcing explicit thrust boundaries and rudder angle constraints, this method better aligns with actual ship maneuvering capabilities. Nevertheless, the absence of a unified task coordinator between collision avoidance and cruising modules may induce conflicting control actions. These observations indicate that existing control methods still struggle to reconcile collision avoidance, multi-task coordination, and compatibility with physical constraints. Overall, these studies demonstrate substantial progress in perception, guidance, and control for unmanned and intelligent surface vessels. However, an integrated optimization-based control framework that can simultaneously handle cruise tracking, dynamic collision avoidance, and realistic actuator constraints for intelligent tugboats in congested port environments is still lacking. Beyond the marine domain, optimization and decision-making under uncertainty have been extensively studied in fields such as supply chain design, robust project scheduling, and energy-aware production planning [12–16]. These works show how optimization models can balance multiple conflicting objectives and enforce operational constraints in complex systems. In a similar spirit, the quadratic programming-based cruise control framework proposed in this paper adopts an optimization perspective, but focuses on real-time control of an intelligent tugboat under dynamic collision-avoidance and actuation constraints.

Motivated by the above literature and current research status, this paper proposes a quadratic programming-based autonomous cruise control method for intelligent tugboats to address the multi-objective control problem in autonomous cruising. The method enables the tugboat to reach the target position with specified speed, heading, and path, within its actuation limits, while strictly avoiding collisions with other vessels. First, a regulation error function is constructed, and desired control inputs are designed using sliding mode control and backstepping to realize cruise control with respect to speed, path, and heading. Second, based on Nagumo’s theorem, a corresponding control strategy is established to avoid collisions between the tugboat and dynamic obstacles. Third, considering the input constraints in practical applications of the tugboat, a comprehensive controller is designed using a quadratic programming approach [17], which can effectively coordinate collision avoidance and cruising tasks under multiple constraints and determine the optimal control strategy. Finally, simulation experiments are conducted to verify the effectiveness of the proposed autonomous cruise control method. Throughout this paper, the term “autonomous cruise control” specifically refers to the closed-loop control layer of an intelligent tugboat that, for a given reference path and desired cruising speed, autonomously computes the thrust commands in surge and sway as well as the yaw moment for the azimuth thrusters. Once the reference is specified, this layer operates without manual steering intervention, while higher-level tasks such as mission assignment and route generation may still be handled by human operators or supervisory systems.

## 2. Problem formulation

**Fig 1:**
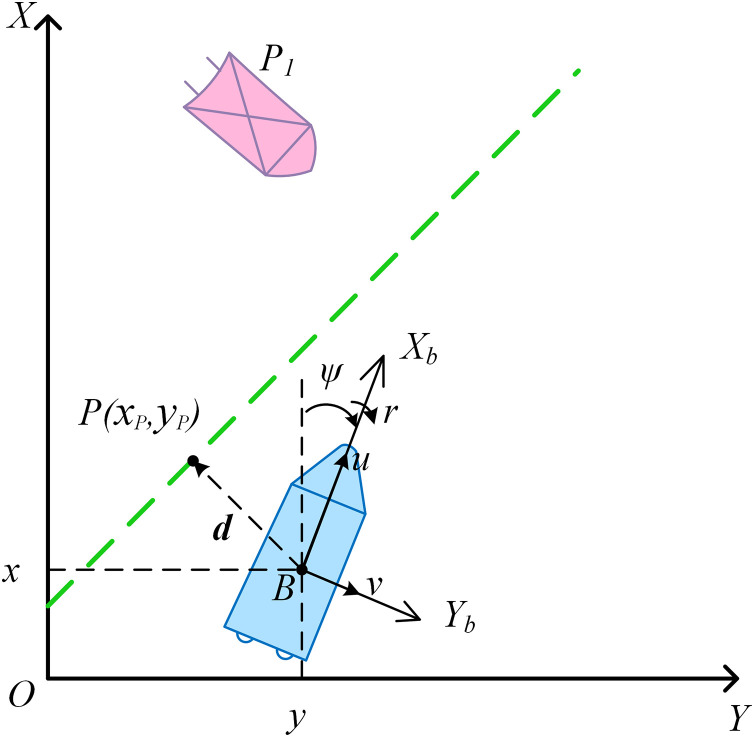
Schematic diagram of autonomous cruising of a tugboat.

Surface vessels typically consider motion in three degrees of freedom (3-DOF) on the horizontal plane [18], To describe the autonomous cruising behavior of an intelligent tugboat, a global coordinate frame XOY and a body-fixed coordinate frame $X_{b}BY_{b}$ are established, as shown in Fig 1, where the blue vessel represents the tugboat and the pink vessel represents an obstacle ship.

The pose vector of the tugboat in the global frame is denoted as $\eta =[x,y,\psi {]}^{T}$, where (x,y) are the position coordinates, and ψ is the heading angle. The velocity vector in the body-fixed frame is given by $\upsilon =[u,v,r{]}^{T}$, where u and v are the surge and sway velocities, respectively, and r is the yaw rate. The kinematic model of the tugboat [19] can be expressed as:


$$\dot{\eta }=R\left(\psi \right)\upsilon$$ (1)

where $\dot{\eta }$ is the time derivative of the pose vector, and $R_{o}\left(\psi \right)=\left[\begin{array}{cc} @cc@cos\psi & -sin\psi \\ sin\psi & cos\psi \end{array}\right]$,$R\left(\psi \right)=\left[\begin{array}{cc} @cc@R_{o}\left(\psi \right) & 0_{2\times 1}\\ 0_{1\times 2} & 1 \end{array}\right]$ is the rotationmatrix. To enable dynamic obstacle avoidance during cruising, the real-time positions and velocities of surrounding vessels must be updated. The pose and velocity vectors of an obstacle ship are denoted as $\eta_{l}=[x_{l},y_{l},\psi_{l}{]}^{T}$ and $\upsilon_{l}=[u_{l},v_{l},r_{l}{]}^{T}$.

The dynamic model of the intelligent tugboat [20] is given by:


$$M\dot{\upsilon }=-C\left(\upsilon \right)\upsilon -D\left(\upsilon \right)\upsilon +\tau$$ (2)

where $M=\left[\begin{array}{ccc} @ccc@m_{11} & 0 & 0\\ 0 & m_{22} & m_{23}\\ 0 & m_{32} & m_{33} \end{array}\right]$ is the inertia matrix,$\dot{\upsilon }$ is the derivative of υ with respect to time, $C\left(\upsilon \right)=\left[\begin{array}{ccc} @ccc@0 & 0 & -m_{11}v-m_{23}r\\ 0 & 0 & -m_{11}u\\ m_{11}v+m_{23}r & m_{11}u & 0 \end{array}\right]$ is the Coriolis-centripetal matrix, $D\left(\upsilon \right)=\left[\begin{array}{ccc} @ccc@d_{11}\left(\upsilon \right) & 0 & 0\\ 0 & d_{22}\left(\upsilon \right) & d_{23}\left(\upsilon \right)\\ 0 & d_{32}\left(\upsilon \right) & d_{33}\left(\upsilon \right) \end{array}\right]$ is thedamping matrix, $m_{11}=m-X_{\dot{u}}$, $m_{22}=m-Y_{\dot{\nu }}$, $m_{32}=mx_{g}-N_{\dot{\nu }}$, $m_{23}=mx_{g}Y_{\dot{r}}$, $m_{33}=I_{z}-N_{\dot{r}}$, $d_{11}\left(\upsilon \right)=-X_{u}-X_{\left|u\right|u}\left|u\right|-X_{uuu}u^{\text{2}}$, $d_{22}\left(\upsilon \right)=-Y_{\nu }-Y_{\left|\nu \right|\nu }\left|v\right|-Y_{\left|r\right|\nu }\left|r\right|$, $d_{23}\left(\upsilon \right)=-Y_{r}-Y_{\left|v\right|r}\left|v\right|-Y_{\left|r\right|r}\left|r\right|$, $d_{32}\left(\upsilon \right)=-N_{\nu }-N_{\left|\nu \right|\nu }\left|v\right|-N_{\left|r\right|\nu }\left|r\right|$, $d_{33}\left(\upsilon \right)=-N_{r}-N_{\left|v\right|r}\left|v\right|-N_{\left|r\right|r}\left|r\right|$, m is the mass, $I_{z}$ is the moment of inertia, $x_{g}$ is the distance between the center of rotation and the center of gravity, $X_{*}$、$Y_{*}$ and $N_{*}$ are the hydrodynamic derivatives, and $\tau ={\left[\tau_{u},\tau_{v},\tau_{r}\right]}^{T}$ is the full-actuated control input vector, consisting of the surge force $\tau_{u}$, sway force $\tau_{v}$, and yaw moment $\tau_{r}$ [21].

In this study, it is assumed that a higher-level guidance or planning module has already generated a smooth reference path $L_{d}$ and a desired cruising speed $u_{d}$ for the intelligent tugboat. Based on these given references, the autonomous cruise control layer designed in this paper is responsible for computing feasible surge, sway, and yaw moment commands while satisfying the following objectives simultaneously:

(1) **Cruise Control Objective:** Under feasible conditions, the tugboat should be controlled to follow the desired speed $u_{d}$, path $L_{d}$, and heading angle $\psi_{d}$, thereby enhancing operational efficiency and reducing navigational risk. In the global frame, the reference path $L_{\text{d}}$ is defined as:

$$ax_{p}+by_{p}+c=0$$ (3)

where $\left(x_{p},y_{p}\right)$ is the foot of the perpendicular from the tugboat to the desired path, and a、b and c are the path parameters (depicted as the green dashed line in Fig 1. The foot-point vector from the tugboat to the desired path is computed based on Equation (3) as follows:


$$d={\left[-\frac{a(ax+by+c)}{a^{\text{2}}+b^{\text{2}}},-\frac{b(ax+by+c)}{a^{\text{2}}+b^{\text{2}}}\right]}^{T}$$ (4)

To analyze the motion state conveniently, the vector d is transformed into $X_{b}BY_{b}$ as:


$$d_{b}=\overset{T}{\underset{o}{R}}\left(\psi \right)d$$ (5)

The desired heading angle $\psi_{d}=arctan(-\frac{a}{b})$, here arctan(·) is the inverse tangent function.

(2) **Collision Avoidance Constraint:** The Euclidean distance between the tugboat and the obstacle ship is defined as:

$$l=\sqrt{{\left(x-x_{l}\right)}^{\text{2}}+{\left(y-y_{l}\right)}^{\text{2}}}$$ (6)

This distance must always be greater than a predefined minimum safety distance $l_{\text{0}}$, that is, $l-l_{\text{0}}>0$, The safety distance is a fixed value manually set based on the characteristics of the vessels, ensuring that the tugboat can safely avoid dynamic obstacles.

(3) **Input Constraints:** The final control command must always remain within the safe operational range of the azimuth thrusters. Specifically, the thrust value τ must lie between the maximum thrust $\tau_{max}$ and the minimum thrust $\tau_{min}$.

## 3. Control strategy design

Intelligent tugboats are typically equipped with omnidirectional thrusters, allowing thrust to be allocated in arbitrary directions and offering greater flexibility in controller design. Fully exploiting this omnidirectional propulsion capability and improving input coordination are crucial for achieving multi-objective control optimization. The proposed control strategy consists of the following modules: speed control, path-following control, heading control, collision avoidance control, and multi-objective coordination control.

### 3.1. Speed control

During autonomous cruise, the cruising speed of the tugboat must be adjusted according to different task requirements. For example, the tugboat may need to operate at a low speed in port areas, or maintain a relatively constant cruising speed during long-distance transit. Therefore, the objective of speed control is to ensure that, under feasible conditions, the surge velocity of the tugboat remains steadily aligned with the desired speed throughout the cruising process. To regulate the thrust of the propulsion system based on the speed error and bring the actual velocity as close as possible to the target value, the speed tracking error is defined as:


$$e_{u}=u-u_{d}$$ (7)

where $u_{d}>0$.

To ensure the asymptotic stability of the speed tracking error $e_{u}$, the desired control input for cruise speed regulation should satisfy:


$${\dot{e}}_{u}=\dot{u}=-k_{u}e_{u}$$ (8)

where ${\dot{e}}_{u}$ and $\dot{u}$ denote the time derivatives of $e_{u}$ and u, respectively, and $k_{u}>0$ is a convergence rate tuning parameter.

### 3.2. Path-following control

During autonomous cruising tasks, to avoid excessive yaw deviation and reduce navigational risks, the intelligent tugboat must follow a predefined reference path $L_{\text{d}}$. From Equation (5), define $d_{b}={\left[e_{x},e_{y}\right]}^{T}$. On this basis, the longitudinal and lateral deviation components of the tugboat with respect to the reference path, denoted by $e_{x}$ and $e_{y}$, are calculated. These two error components represent the tugboat’s deviation along the direction of the path and perpendicular to it, respectively, and are expressed as follows:


$$e_{x}=-\frac{\left(ax+by+c)(acos\psi +bsin\psi \right)}{a^{\text{2}}+b^{\text{2}}}$$ (9)

$$e_{y}=\frac{\left(ax+by+c)(asin\psi -bcos\psi \right)}{a^{\text{2}}+b^{\text{2}}}$$ (10)

The lateral deviation $e_{y}$ is used as the regulation error for path following.

According to the sliding mode control principle, a new regulation error is defined as: According to the sliding mode control principle, a new regulation error $\sigma_{\nu }={\dot{e}}_{y}+k_{y}e_{y}$ is defined, where ${\dot{e}}_{y}$ is the time derivative of $e_{y}$, and $k_{y}>0$ is a convergence rate tuning parameter. To ensure that $\sigma_{\nu }$ is asymptotically stable, the path-following control input should satisfy:


$${\dot{\sigma }}_{\nu }={\ddot{e}}_{y}+k_{y}{\dot{e}}_{y}=-k_{\nu }\sigma_{\nu }$$ (11)

where ${\dot{\sigma }}_{\nu }$ and ${\ddot{e}}_{y}$ denote the time derivatives of $\sigma_{\nu }$ and ${\dot{e}}_{y}$, respectively, and $k_{\nu }>0$ is the proportional feedback gain that regulates the convergence rate.

### 3.3. Heading control

When performing cruising missions, it is critical for tugboats to precisely adjust their heading to maintain strict alignment with the prescribed course after reaching the target navigation route, whenever operational conditions permit. The heading angle adjustment error is defined as:


$$e_{\psi }=\psi -\psi_{d}$$ (12)

According to the principle of sliding mode control, a new regulation error is further defined as $\sigma_{r}={\dot{e}}_{\psi }+k_{\psi }e_{\psi }$, where ${\dot{e}}_{\psi }$ is the time derivative of $e_{\psi }$, and $k_{\psi }>0$ is a convergence rate tuning parameter. To ensure the asymptotic stability of $\sigma_{r}$, the desired heading control input is designed to satisfy:


$${\dot{\sigma }}_{r}=\dot{r}+k_{\psi }r=-k_{r}\sigma_{r}$$ (13)

where ${\dot{\sigma }}_{r}$ and $\dot{r}$ are the time derivatives of $\sigma_{r}$ and r, respectively, and $k_{r}>0$ is a positive feedback gain used to adjust the convergence rate.

To simultaneously accomplish the aforementioned path-following, constant-speed, and heading-regulation tasks, the desired control input $\tau_{r}$ for the intelligent tugboat is formulated in vector form based on Equations (2), (8), (11), and (13) as follows:


$$\tau_{r}=C\left(\upsilon \right)\upsilon +D\left(\upsilon \right)\upsilon -MG^{-1}(f_{a1}+f_{c1})$$ (14)

where $G=\left[\begin{array}{ccc} @ccc@1 & 0 & 0\\ g_{\text{1}} & -g_{\text{2}} & -e_{x}\\ 0 & 0 & 1 \end{array}\right]$, $g_{\text{1}}=\frac{\left(asin\psi -bcos\psi )(acos\psi +bsin\psi \right)}{a^{\text{2}}+b^{\text{2}}}$, $g_{\text{2}}=\text{\textbackslash{}hspace\{1em}\}\frac{(asin\psi -bcos\psi {)}^{\text{2}}}{a^{\text{2}}+b^{\text{2}}}$, $f_{a1}=[0,\delta_{\text{1}}+k_{y}{\dot{e}}_{y},k_{\psi }r{]}^{T}$, $\delta_{\text{1}}=\left(\frac{\left(\begin{array}{c} @l@(a^{\text{2}}-b^{\text{2}})cos\psi \\ +2absin\psi \end{array}\right)\dot{x}-\left(\begin{array}{c} @l@(a^{\text{2}}-b^{\text{2}})sin\psi \\ -2abcos\psi \end{array}\right)\dot{y}}{a^{\text{2}}+b^{\text{2}}}-{\dot{e}}_{x}\right)r$, $f_{c1}=[k_{u}e_{u},k_{v}\sigma_{v},k_{r}\sigma_{r}{]}^{T}$.

When the heading of the tugboat is perpendicular to the desired path such that |G|=0, to avoid singularities in the controller defined by Equation (11), the expected control input at this moment is switched to a proportional controller. The control input is then given by:


$$\tau_{r}=-f_{c1}$$ (15)

where $f_{c1}=[k_{u}e_{u},k_{v}\sigma_{v},k_{r}\sigma_{r}{]}^{T}$.

### 3.4. CBF-based collision avoidance

When encountering obstacles during autonomous cruising, the intelligent tugboat must strictly comply with collision avoidance constraints. The design of collision avoidance control must consider not only speed and heading adjustments, but also adaptively adjust strategies based on environmental changes to avoid collisions with other vessels and ensure navigational safety. To ensure that the tugboat maintains a safe distance from obstacles at all times, the following control barrier function [22] is designed:


$$h=l-l_{\text{0}}$$ (16)

where $l_{\text{0}}>0$ is the predefined minimum safe distance.

To ensure the forward invariance of h≥0, a new control barrier function is constructed based on Nagumo’s theorem as:


$$f=\dot{h}+k_{h}h$$ (17)

where $\dot{h}$ is the time derivative of h, and $k_{h}$ collision avoidance tuning parameter for safety.

To further guarantee the forward invariance of f≥0, the tugboat’s control input should satisfy:


$$\dot{f}=\ddot{h}+k_{h}\dot{h}\geq -k_{f}f$$ (18)

where $\dot{f}$ and $\ddot{h}$ are the time derivatives of f and $\dot{h}$, respectively, and $k_{f}>0$ is a collision avoidance tuning parameter.

To simplify controller implementation, Equation (18) is rewritten as a vector-matrix product form:


$$f_{a2}+g^{T}\tau \geq -f_{c2}$$ (19)

where $f_{a2}=-g^{T}\left(C\left(\upsilon \right)\upsilon +D\left(\upsilon \right)\upsilon \right)+\delta_{\text{2}}+k_{h}\dot{h}$, $g=M^{-T}g_{\text{5}}$, $\delta_{\text{2}}=(\dot{x}-{\dot{x}}_{l}{)}^{\text{2}}+(\dot{y}-{\dot{y}}_{l}{)}^{\text{2}}-(x-x_{l})({\ddot{x}}_{l}+\dot{y}r)-(y-y_{l})({\ddot{y}}_{l}-\dot{x}r)$, $g_{\text{5}}=[g_{\text{3}},-g_{\text{4}},0{]}^{T}$,$g_{\text{3}}=\left(\begin{array}{c} @c@x-x_{l} \end{array}\right)cos\psi +\left(\begin{array}{c} @c@y-y_{l} \end{array}\right)sin\psi$, $g_{\text{4}}=(x-x_{l})sin\psi -(y-y_{l})cos\psi$, $f_{c2}=k_{f}f$.

### 3.5. Integrated controller based on quadratic programming

In real-world navigation, the control inputs to the tugboat’s azimuth thrusters are subject to fixed physical constraints. If the control commands exceed these physical limits, not only will the expected control effect fail to materialize, but the thrusters may also suffer damage, posing serious safety risks. To ensure that the control output remains within the thrust capabilities of the azimuth propulsion system, strict bounds must be imposed on the control input as:


$$\tau_{min}\leq \tau \leq \tau_{max}$$ (20)

Based on the actuator’s physical constraints, the controller must also ensure strict satisfaction of the non-negotiable hard constraint imposed by collision avoidance. Under this prerequisite, it should further seek to fulfill the performance requirements of speed, heading, and path tracking as much as possible. In other words, the designed control input must strictly satisfy constraints (19) and (20), while making the control output approximate Equation (14) as closely as possible. To address this multi-objective constrained control problem, a quadratic programming approach is adopted. The quadratic programming formulation determines the optimal control input that minimizes the deviation from the nominal control command while satisfying all constraints. The optimization problem is expressed as:


$$\begin{array}{c} \begin{array}{c} \tau^{*}=arg\underset{\tau \in {\mathbb{R}}^{\text{3}}}{min}{\left(M^{-1}(\tau -\tau_{r})\right)}^{T}H\left(M^{-1}(\tau -\tau_{r})\right)\\ \text{s}.\text{t}.f_{a2}+g^{T}\tau \geq -f_{c2}\\ \tau_{min}\leq \tau \leq \tau_{max} \end{array} \end{array}$$ (21)

where $H=diag\left(h_{\text{1}},h_{\text{2}},h_{\text{3}}\right)$ is a positive definite weighting matrix, and $h_{\text{1}},h_{\text{2}},h_{\text{3}}>0$ are tuning parameters used to adjust the relative importance of different control objectives. Quadratic programming effectively handles the challenge of satisfying multiple objectives and constraints simultaneously, while offering strong real-time solvability for dynamic maritime environments. The control problem is formulated as a structured optimization task, enabling real-time thrust adjustment based on the current navigation state. This ensures strict safety constraint satisfaction and coordinated control of heading, speed, and path.

### 3.6. Stability analysis

**Theorem 1.** *For the autonomous cruise control problem of tugboats, under the control law designed in this paper and the quadratic programming framework that incorporates thrust saturation and collision avoidance constraints through control barrier functions (CBFs), the closed-loop system possesses the following properties. The control input generated by the quadratic programming strictly satisfies the thrust and safety constraints at all times, thereby ensuring that the feasible set is forward invariant and the vessel remains within the safe operating region. Moreover, the tracking error dynamics are stabilized in the sense that, when no constraints are activated, the errors asymptotically converge to the origin, while in the presence of active constraints the errors remain uniformly ultimately bounded, with the ultimate bound determined by the control parameters and the magnitude of the constraint-induced correction.*

**Proof of Theorem 1.** To analyze the closed-loop stability of the proposed controller under different operating scenarios, the following Lyapunov candidate function is constructed:


$$V=\frac{\text{1}}{\text{2}}{\tilde{e}}^{T}\tilde{e}$$ (22)

where $\tilde{e}={\left[e_{u},\sigma_{\nu },\sigma_{r}\right]}^{T}$ denotes the composite error vector.

By the design of the quadratic program, all control inputs remain within the hard constraint set, and thus both the thrust saturation constraints and the CBF-based collision avoidance constraints are always satisfied. This corresponds to the first part of the theorem. On this basis, we now consider the convergence of the tracking errors.

When the planned control input satisfies$\tau =\tau_{r}$, two cases arise:

**Case 1:** When|G|=0, from Eq. (14), we have


$$\left|G\left(\psi \right)\right|=-g_{\text{2}}\left(\psi \right)=-\frac{(asin\psi -bcos\psi {)}^{\text{2}}}{a^{\text{2}}+b^{\text{2}}}={cos}^{\text{2}}\left(\psi -\psi_{d}\right)$$ (23)

which indicates


$$\left|G\right|=0⟺cos\left(\psi -\psi_{d}\right)=0⟺\psi -\psi_{d}=\frac{\pi }{\text{2}}+n\pi ,n\in \mathbb{Z}.$$ (24)

Namely, the geometric matrix becomes non-invertible when the tugboat’s heading is perpendicular to the desired heading. Under this condition, the yaw fallback control $\tau_{r}=-k_{r}\sigma_{r}$ yields


$$\underset{t\to \infty }{lim\text{\textbackslash{}hspace\{0.33em}}sup\;\}\;∥\tilde{e}\left(t\right)∥\leq R_{\tilde{e}}≜\frac{\sqrt{\text{2}}\gamma }{\sqrt{\alpha \theta }}\Delta_{max}$$ (25)

Rearranging, we obtain


$$\dot{r}=-\frac{k_{r}}{m_{33}}r-\frac{k_{r}k_{\psi }}{m_{33}}\left(\psi -\psi_{d}\right)-\frac{m_{23}}{m_{33}}\dot{v}-\frac{c_{\text{3}}\left(\nu \right)+d_{\text{3}}\left(\nu \right)}{m_{33}}$$ (26)

Which implies


$$\dot{r}\;≢\;0\Rightarrow ≢\;0\;\Rightarrow \psi \;≢\;\psi_{d}+\frac{\pi }{\text{2}}+n\pi$$ (27)

In other words, $\psi =\psi_{d}+\frac{\pi }{\text{2}}+n\pi$ is not an attractor.

**Case 2:** When |G|≠0, taking time derivative of V and substituting in Equations (14) into the result yields


$$\dot{V}=-{\tilde{e}}^{⊤}K\tilde{e}\leq -k_{min}V$$ (28)

where $K=diag\left(k_{u},k_{\nu },k_{r}\right)$, $k_{min}=min\left\{2k_{u},2k_{\nu },2k_{r}\right\}$.

In light of the comparison principle, we have from Equations (22) that


$$V\left(t\right)\leq V\left(0\right)e^{-2\alpha t},\text{\textbackslash{}hspace\{1em}\}\forall t\geq 0$$ (29)

Where $\alpha =min\left\{k_{u},k_{\nu },k_{r}\right\}>0$.

i.e., V(t) is asymptotically stable, and we further conclude that $\tilde{e}$ converges to zero asymptotically.

Furthermore, since $\sigma_{\nu }={\dot{e}}_{y}+k_{\nu }e_{y}$, when $\sigma_{\nu }\to 0$, the pair $\left(e_{y},{\dot{e}}_{y}\right)$ evolves according to a stable first-order linear system. Consequently, it follows that $e_{y}\left(t\right)\to 0$ ast→∞. Similarly, from $\sigma_{r}={\dot{e}}_{\psi }+k_{\psi }e_{\psi }$, the convergence $\sigma_{r}\to 0$, implies that $e_{\psi }\left(t\right)\to 0$. Therefore, not only the auxiliary errors $\sigma_{\nu }$ and $\sigma_{r}$ converge to zero, but also the position error $e_{y}$ and the heading error $e_{\psi }$ asymptotically converge to zero.

When the planned control input satisfies $\tau =\tau_{r}$, thrust saturation or collision-avoidance constraints are triggered, the actual control input is obtained by solving a quadratic programming optimization problem:


$$\begin{array}{c} \tau^{*}=arg\underset{\tau \in {\mathbb{R}}^{\text{3}}}{min}{\left(M^{-1}\left(\tau -\tau_{r}\right)\right)}^{T}H\left(M^{-1}\left(\tau -\tau_{r}\right)\right)\\ s.t.\text{\textbackslash{}hspace\{1em}\}constrains.\text{\textbackslash{}hspace\{0.33em}\;\;\} \end{array}$$ (30)

Define the control deviation as:


$$\Delta \tau =\tau^{*}-\tau_{r}$$ (31)

In this case, the derivative of the Lyapunov function is:


$$\dot{V}\leq -k_{min}∥\tilde{e}{∥}^{\text{2}}+\gamma ∥\tilde{e}∥\;∥\;\Delta \tau ∥$$ (32)

$$\gamma =∥D∥\;=∥M^{-1}∥$$ (33)

Applying Young’s inequality to the second term, for any $0<\theta <2k_{min}$, we have:


$$\gamma ∥\tilde{e}∥\;∥\;\Delta \tau ∥\leq \theta ∥\tilde{e}{∥}^{\text{2}}+\frac{\gamma^{\text{2}}}{4\theta }∥\;\Delta \tau {∥}^{\text{2}}$$ (34)

Substituting this into the above yields:


$$\dot{V}\leq -\alpha V+\beta \left(t\right)$$ (35)

$$\alpha =2\left(k_{min}-\frac{\theta }{\text{2}}\right)>0$$ (36)

$$\beta \left(t\right)=\frac{\gamma^{\text{2}}}{\theta }∥\;\Delta \tau \left(t\right){∥}^{\text{2}}$$ (37)

In this case,


$$\beta \left(t\right)\leq \beta ≜\frac{\gamma^{\text{2}}}{\theta }\overset{\text{2}}{\underset{max}{\Delta }}$$ (38)

The comparison lemma yields:


$$\underset{t\to \infty }{limsup}\;V\left(t\right)\leq \frac{\overset{―}{\beta }}{\alpha }$$ (39)

From $V=\frac{\text{1}}{\text{2}}∥\tilde{e}{∥}^{\text{2}}$, it follows that:


$$\underset{t\to \infty }{lim\text{\textbackslash{}hspace\{0.33em}}sup\;\}\;∥\tilde{e}\left(t\right)∥\leq R_{\tilde{e}}≜\frac{\sqrt{\text{2}}\gamma }{\sqrt{\alpha \theta }}\Delta_{max}$$ (40)

Furthermore, by considering the first channel,


$${\dot{e}}_{y}+k_{y}e_{y}=\sigma_{\nu }$$ (41)

$${\dot{e}}_{\psi }+k_{\psi }e_{\psi }=\sigma_{r}$$ (42)

We obtain:


$$lim\text{\textbackslash{}hspace\{0.33em}\}sup\left|e_{u}\right|\leq R_{\tilde{e}}$$ (43)

$$lim\text{\textbackslash{}hspace\{0.33em}\}sup\left|e_{y}\right|\leq \frac{\text{1}}{k_{y}}R_{\tilde{e}}$$ (44)

$$lim\text{\textbackslash{}hspace\{0.33em}\}sup\left|e_{\psi }\right|\leq \frac{\text{1}}{k_{\psi }}R_{\tilde{e}}$$ (45)

Therefore, the original error is uniformly ultimately bounded (UUB), and the final bound radius is proportional to $\Delta_{max}$.

## 4. Simulation study and results

### 4.1. Simulation conditions

To verify the effectiveness and superiority of the proposed quadratic programming-based autonomous cruise control method for intelligent tugboats, a comprehensive simulation study is conducted in MATLAB, along with the design of comparative experiments for benchmark analysis. In Comparison Method A, only constant-speed and heading control are applied to reach the target position. In Comparison Method B, collision avoidance with other vessels is introduced based on the APF, in addition to constant-speed and heading control. The CyberShip II tugboat is adopted as the motion model [23], and its parameters are listed in Table 1.

**Table 1:** Main model parameters of the CyberShip II.

Parameter	Value	Unit	Parameter	Value	Unit	Parameter	Value	Unit
$m_{11}$	25.8	kg	$Y_{v}$	-0.8612	kg/s	$N_{\dot{v}}$	0.0	kg ⋅ m
$m_{22}$	33.8	kg	$Y_{\dot{v}}$	-10.0	kg	$N_{\dot{r}}$	-1.0	kg ⋅ m^2^
$m_{23}$	1.0948	kg	$Y_{\left|v\right|v}$	-36.2823	kg/m	$N_{\left|v\right|v}$	5.0437	kg
$m_{32}$	1.0948	kg	$Y_{\left|r\right|v}$	-8.05	kg/rad	$N_{\left|r\right|v}$	0.13	kg ⋅ m/rad
$m_{33}$	2.76	kg	$Y_{\left|v\right|r}$	-0.845	kg/rad	$N_{r}$	-1.9	kg ⋅ m^2^/(s ⋅ rad)
$X_{u}$	-0.7225	kg/s	$Y_{\left|r\right|r}$	-3.45	kg ⋅ m/rad^2^	$N_{\left|v\right|r}$	0.08	kg ⋅ m/rad
$X_{\dot{u}}$	-2.0	kg	$Y_{r}$	0.1079	kg ⋅ m/(s ⋅ rad)	$N_{\left|r\right|r}$	-0.754	kg ⋅ m^2^/rad^2^
$X_{\left|u\right|u}$	-1.3274	kg/m	$Y_{\dot{r}}$	0.0	kg ⋅ m			
$X_{uuu}$	-5.8664	kg ⋅ s/m^2^	$N_{v}$	0.1052	kg ⋅ m/s			

During the simulation, the initial state of the tugboat is set to $\eta \left(0\right)={\left[5,0,\frac{\pi }{\text{3}}\right]}^{T}$ and $\nu \left(0\right)=[3,0,0{]}^{T}$, The path parameters for path-following are a=1, b=−1, c=0, the desired cruising speed for speed control is $u_{d}=4$, the desired heading angle for heading control is$\psi_{d}=0.7854$, the thrust input is constrained within the bounds $\tau_{max}=[400,80,165{]}^{T}$ and $\tau_{min}=[-400,-80,-165{]}^{T}$, the minimum safe distance from the obstacle vessel is $l_{\text{0}}=10$, and the control gains and tuning parameters are summarized in Table 2.

**Table 2:** Control parameters.

Parameter	$k_{y}$	$k_{v}$	$k_{u}$	$k_{\psi }$	$k_{r}$	$k_{h}$	$k_{f}$	$h_{\text{1}}$	$h_{\text{2}}$	$h_{\text{3}}$
**Value**	1	2	1	1	2	1	2	1	0.1	1

### 4.2. Simulation results and comparison

**Fig 2:**
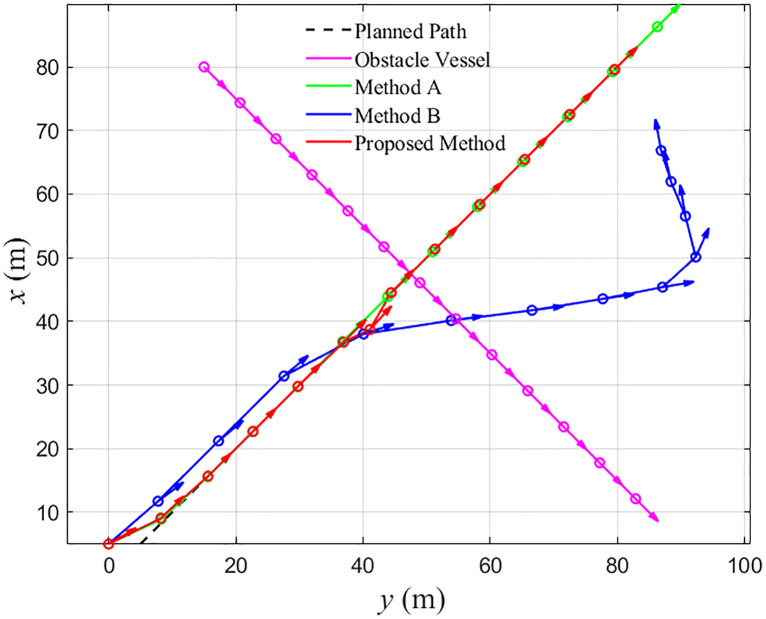
Schematic diagram of the tugboat’s trajectory.

**Fig 3:**
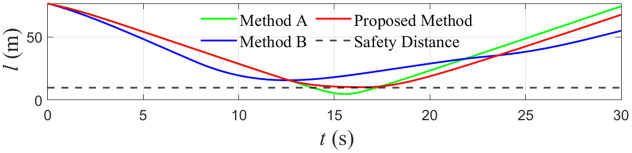
The distance between the tugboat and the barrier boat.

**Fig 4:**
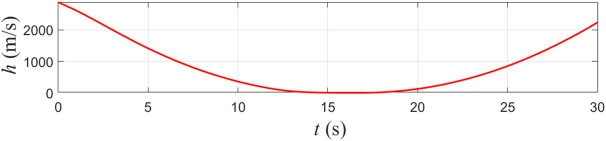
Control Barrier Function *h.*

**Fig 5:**
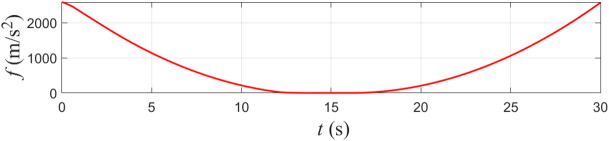
Control Barrier Function *f.*

**Fig 6:**
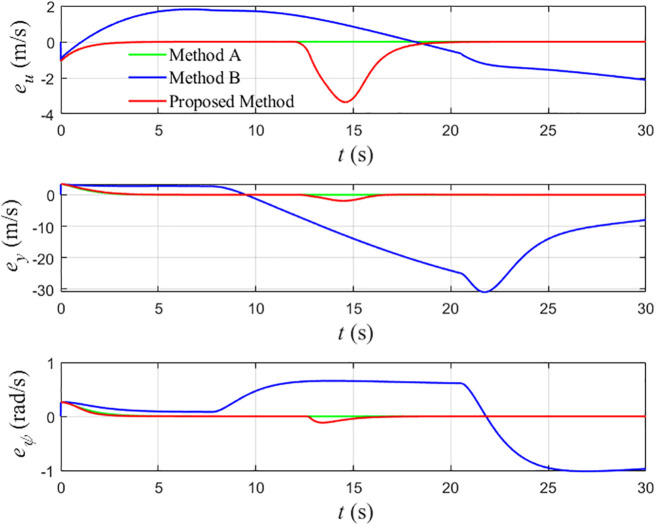
Comparison chart of the error change of the three methods.

**Fig 7:**
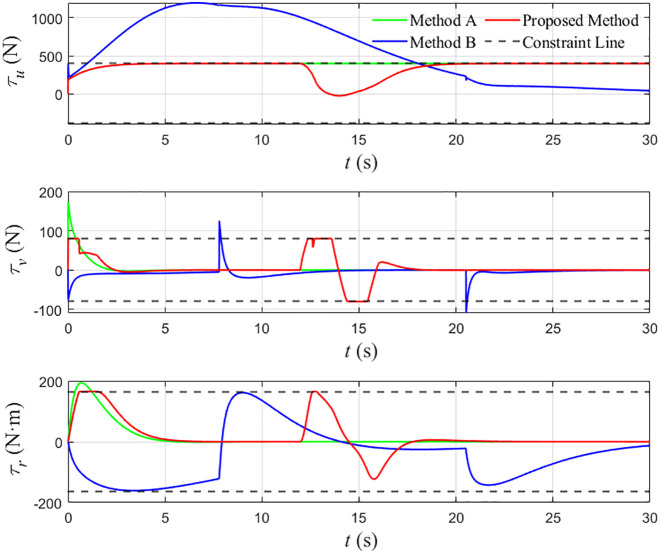
Comparison chart of thrust changes of the three methods.

Figs 2-7 and Table 3 compare the three control schemes in an autonomous cruising and collision-avoidance scenario. As shown in Fig 2, the tugboat is required to track a prescribed reference trajectory while passing a moving obstacle vessel. Under Comparison Method A, the tugboat adjusts its heading toward the reference and eventually reaches the target region, but the resulting trajectory passes very close to the obstacle vessel. Comparison Method B initiates the avoidance maneuver earlier and turns away from the obstacle at a relatively large distance; however, the vessel deviates substantially from the reference and only returns to it after a long transient. In contrast, the proposed method generates a smoother detour around the obstacle and limits the overall deviation from the reference trajectory.

**Table 3:** Safety margin and thrust demand for the three control schemes.

Scheme Method	MinDist (m)	Max$\left|\tau_{\text{u}}\right|$ (N)	Max$\left|\tau_{\text{v}}\right|$ (N)	Max$\left|\tau_{\text{r}}\right|$ (N·m)
**Method A**	4.789	399.6	174.3	193.9
**Method B**	15.685	1192.7	125.3	161.5
**Proposed**	10.215	399.6	80.0	165.0

The safety performance of the three controllers is quantified in Fig 3 and Table 3. For Comparison Method A, the minimum distance between the tugboat and the obstacle vessel is 4.79 m, which is far below the prescribed safety distance $l_{\text{0}}=10$ m and therefore indicates a high collision risk. Comparison Method B maintains the largest clearance, with a minimum distance of 15.69 m, but this comes at the expense of an unnecessarily conservative maneuver. For the proposed controller, the minimum distance is 10.22 m, slightly above the safety threshold. This demonstrates that the control barrier function–based constraint in the QP keeps the vessel just outside the unsafe region, providing guaranteed collision avoidance without excessive conservatism.

The evolution of the control barrier functions h and f for the proposed controller is shown in Fig 4 and Fig 5. Both functions remain strictly positive over the entire simulation horizon, confirming that the safety set defined by the distance constraint is forward invariant. In other words, starting from a safe initial condition, the closed-loop system is prevented from entering the unsafe region surrounding the obstacle vessel.

Fig 6 compares the tracking errors in surge speed, cross-track position, and heading angle. Because Comparison Method A does not incorporate any collision-avoidance mechanism, it achieves the smallest steady-state errors when no obstacle interaction is present. Comparison Method B, although capable of avoiding collision, exhibits the largest cross-track and heading errors due to its conservative avoidance strategy and slow recovery toward the reference. The proposed controller yields intermediate error levels: the tracking errors increase during the avoidance maneuver but remain much smaller than those of Comparison Method B and decay rapidly once the obstacle has been passed. This indicates that the proposed scheme achieves a balanced compromise between tracking performance and safety.

The thrust demands of the three schemes are presented in Fig 7 and Table 3. For Comparison Method B, the surge force $\tau_{u}$ reaches approximately 1193 N, which significantly exceeds the actuator limit and renders this strategy infeasible for practical implementation. Comparison Method A satisfies the surge-force bound but generates peak sway force and yaw moment of 174.3 N and 193.9 N·m, respectively, both beyond their admissible ranges. By contrast, the proposed QP-based controller keeps all three control inputs within their predefined bounds while still ensuring collision avoidance and acceptable tracking accuracy. Overall, the simulation study shows that the proposed method can simultaneously satisfy safety and actuation constraints and maintain good cruising performance, which is crucial for the practical deployment of autonomous tugboats in congested port environments.

## 5. Conclusions

This paper presents a quadratic programming-based autonomous cruise control method for intelligent tugboats to simultaneously address speed tracking, path following, heading regulation, collision avoidance, and input constraints. By constructing regulation errors and designing desired control inputs via sliding mode and backstepping techniques, the tugboat achieves accurate cruise control. A control barrier function based on the relative distance to obstacles is introduced to convert safety constraints into input constraints. Thrust limits are enforced to ensure physical feasibility, and a quadratic programming -based controller coordinates all objectives to compute optimal control inputs. Simulation results confirm that the proposed method enables safe and efficient cruise with minimal deviation, showing strong potential for practical application. Future work will consider environmental disturbances and multi-vessel cooperation to enhance robustness and adaptability.

## References

[1] Liu J, Xu C, Li S, Dong Z, Liu J, Zhao Y, et al. Towards future autonomous tugs: Design and implementation of an intelligent escort control system validated by sea trials. Advanced Engineering Informatics. 2025;65:103116. doi: 10.1016/j.aei.2025.103116

[2] Liu H, Wang A, Han B, Li T, Wang D, Peng Z. Safety-critical anti-disturbance control of tugs for collaborative berthing. Ocean Engineering. 2024;312:118972. doi: 10.1016/j.oceaneng.2024.118972

[3] Liu J, Yang F, Xie L, Li S, Wang T. Research on virtual simulation testing technology for intelligent navigation collision avoidance decision-making and planning. Journal of System Simulation. 2024;36(8): 1780–9.

[4] Zhong W, Luo Y, Lu D, Fengo Y, Huang X, Chen C. Design and implementation of unmanned boat communication navigation control system. Shipbuilding of China. 2018;59(1):207–15.

[5] Yu L. The application of deep learning technology in trajectory control systems. Ship Science and Technology. 2024;46(10):174–7.

[6] Zhang R, Liu Y, Anderlini E. Robust trajectory tracking control for unmanned surface vessels under motion constraints and environmental disturbances. Proceedings of the Institution of Mechanical Engineers, Part M: Journal of Engineering for the Maritime Environment. 2021;236(2):394–411. doi: 10.1177/14750902211039663

[7] Zhao L, Roh M-I. COLREGs-compliant multiship collision avoidance based on deep reinforcement learning. Ocean Engineering. 2019;191:106436. doi: 10.1016/j.oceaneng.2019.106436

[8] Zhang Z, Yang N, Yang Y. Autonomous navigation and collision prediction of port channel based on computer vision and lidar. Sci Rep. 2024;14(1):11300. doi: 10.1038/s41598-024-60327-9 38760377 PMC11101439

[9] Zhang K, Huang L, He Y, Zhao X, Chen J, Huang W. Dynamic intelligent collision avoidance method based on trajectory prediction. Navigation of China. 2023;46(04):20–9.

[10] Huang Y, Chen L, van Gelder PHAJM. Generalized velocity obstacle algorithm for preventing ship collisions at sea. Ocean Engineering. 2019;173:142–56. doi: 10.1016/j.oceaneng.2018.12.053

[11] Menges D, Tengesdal T, Rasheed A. Nonlinear model predictive control for enhanced navigation of autonomous surface vessels. IFAC-PapersOnLine. 2024;58(18):296–302. doi: 10.1016/j.ifacol.2024.09.046

[12] Goli A. Integration of blockchain-enabled closed-loop supply chain and robust product portfolio design. Computers & Industrial Engineering. 2023;179:109211. doi: 10.1016/j.cie.2023.109211

[13] Goli A. Efficient optimization of robust project scheduling for industry 4.0: A hybrid approach based on machine learning and meta-heuristic algorithms. International Journal of Production Economics. 2024;278:109427. doi: 10.1016/j.ijpe.2024.109427

[14] Goli A, Ala A, Hajiaghaei-Keshteli M. Efficient multi-objective meta-heuristic algorithms for energy-aware non-permutation flow-shop scheduling problem. Expert Systems with Applications. 2023;213:119077. doi: 10.1016/j.eswa.2022.119077

[15] Goli A, Ala A, Mirjalili S. A robust possibilistic programming framework for designing an organ transplant supply chain under uncertainty. Ann Oper Res. 2022;328(1):493–530. doi: 10.1007/s10479-022-04829-7

[16] Goli A, Tirkolaee EB. Designing a portfolio-based closed-loop supply chain network for dairy products with a financial approach: Accelerated Benders decomposition algorithm. Computers & Operations Research. 2023;155:106244. doi: 10.1016/j.cor.2023.106244

[17] Rosenberger L, Shen Y, Haunert J-H. Simultaneous selection and displacement of buildings and roads for map generalization via mixed-integer quadratic programming. International Journal of Geographical Information Science. 2025;39(7):1567–96. doi: 10.1080/13658816.2025.2461602

[18] Liu J, Dong Z, Li S, You X, Hu Y. Autonomous berthing control of tugboat based on improved backstepping sliding mode control algorithm. Chinese Journal of Ship Research. 2024;19(1):119–27.

[19] Li M, Liu J, Liu K, Gu J, Chen J, Zhao Y. Simulation and experiment of nonlinear model predictive trajectory tracking control algorithm for a fully actuated unmanned tug. Ship Engineering. 2024;46(S1):257–68.

[20] Sun Q, Yin W. Research on large ship berthing technology based on tugboat autonomous stabilization. Shipbuilding of China. 2023;64(4):176–85.

[21] He H, Wang N, Huang D, Han B. Active vision-based finite-time trajectory-tracking control of an unmanned surface vehicle without direct position measurements. IEEE Trans Intell Transport Syst. 2024;25(9):12151–62. doi: 10.1109/tits.2024.3364770

[22] Ames AD, Xu X, Grizzle JW, Tabuada P. Control barrier function based quadratic programs for safety critical systems. IEEE Trans Automat Contr. 2017;62(8):3861–76. doi: 10.1109/tac.2016.2638961

[23] Wang Z, Qiu C, Dong Z, Cheng S, Zheng L, Chen S. Trajectory tracking of unmanned surface vessels based on robust neural networks and adaptive control. Journal of Marine Science and Engineering. 2025;13(7): 1341.

